# The future fate of Somali upwelling productivity and the implications for fisheries under climate change

**DOI:** 10.1038/s41598-026-55455-3

**Published:** 2026-06-17

**Authors:** Z. L. Jacobs, F. Jebri, J. Bruggeman, C. Baker, F. James, M. Srokosz, A. Yool, A. Loveridge, E. Popova

**Affiliations:** 1 National Oceanography Centre, European Way, Southampton, SO14 3ZH UK; 2 Bolding & Bruggeman ApS, Strandgyden 25, Asperup, 5466 Denmark; 3 MacAlister Elliott & Partners, 56 High Street, Lymington, SO41 9AH UK

**Keywords:** Climate sciences, Ecology, Ecology, Ocean sciences

## Abstract

The Somali upwelling is the strongest upwelling region globally during its seasonal peak. The intense productivity that occurs during the southwest monsoon (May-September) sustains artisanal and industrial fisheries. However, due to its complex structure and seasonality, and the typically coarse spatial resolution of global climate models, understanding its future fate remains a challenge. Using a high-resolution future climate projection and a size-spectrum model (in the absence of reliable fish catch data), we identify key climate stressors and projected changes in higher trophic levels to understand potential future impacts on Somali fisheries. Overall, the productivity generated by the Somali upwelling is projected to decline by the end of the century. Our results show that the inner coastal zone may experience elevated productivity, potentially due to changes in the prevailing winds and Somali Current. This may indicate potential climate refugia and minimal impacts to the artisanal fishing fleet. However, further offshore in the Great Whirl region dominated by small pelagic fish, there is a projected decline in productivity and biomass, which may impact the industrial fishing fleets that may target this area in future. To overcome challenges in understanding the fate of global upwelling systems, high-resolution models must be employed to more accurately simulate individual systems.

**Supplementary Information:**

The online version contains supplementary material available at https://doi.org/10.1038/s41598-026-55455-3.

## Introduction

Coastal upwelling systems are crucial for sustaining both industrial and artisanal fisheries globally, which are vital for the food and livelihood security of coastal populations. These systems are driven by the interactions between winds and boundary currents, and are characterised by elevated nutrient supply, sustaining productive marine ecosystems and fisheries^1–3^. Thus, they play a vital role in global and regional economies^4^. Among the coastal upwelling systems influenced by major ocean currents, the Eastern Boundary Upwelling Systems (EBUSs), including the California, Humboldt, Canary, and Benguela Current systems, are the most prominent globally, receiving extensive attention from the research community^5–7^. Combined, they support 20% of the global fish catch^8^. Less recognised but equally crucial for marine ecosystems and dependent coastal communities are the seasonal upwelling systems. In these systems, active upwelling occurs for only part of the year due to specific regional wind features. Given the critical role of upwelling in supporting marine biodiversity and the blue economy, understanding the response to climate change is imperative. However, the projected response of the strength of upwelling systems and their productivity does not exhibit a globally coherent pattern^8,9^, prompting a detailed examination of individual upwelling systems.

**Fig. 1:**
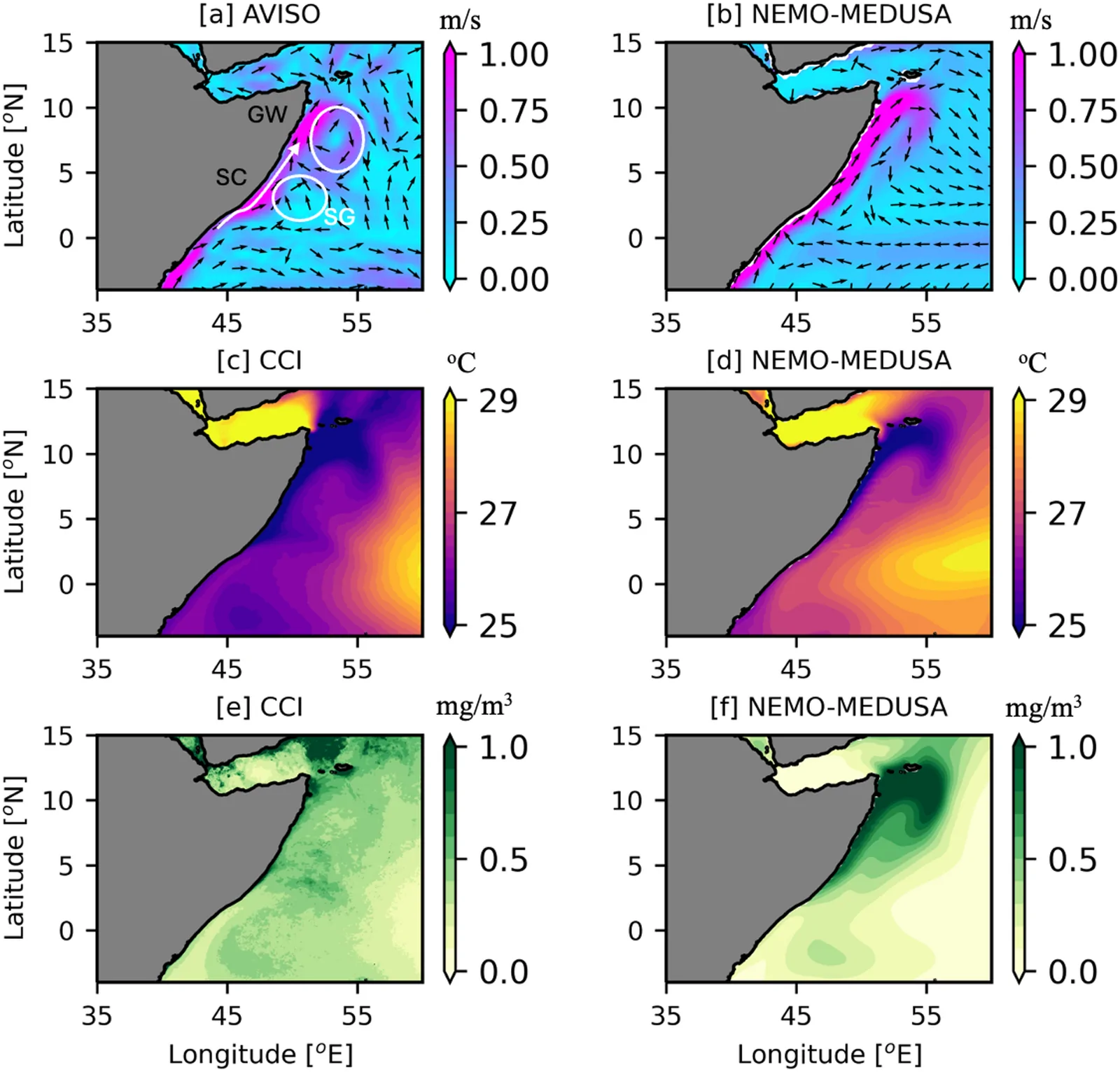
Observed (left) and modelled (right) multidecadal mean (2000–2020) [**a**, **b**] surface currents (m/s), [**c**, **d**] SST (^o^C) and [**e**, **f**] chl-a (mg/m^3^) in July over the wider Somali upwelling region. The grey line and circle on panel [**a**] denote the approximate locations of the Somali Current and Great Whirl respectively.

Here, we focus on the Indian Ocean’s Somali upwelling, which, unlike the EBUSs, occurs off the eastern Somali coast, i.e., at the western basin boundary, and at the height of its occurrence, is the strongest globally^10^. It forms during the southwest monsoon (May-September), when prevailing winds are southwesterly and parallel to the coastline, driven by the Findlater jet^11^, initiating Ekman transport and intense upwelling^12,13^. The jet drives the fast, up to 3 m/s^14^, northward-flowing Somali Current at this time of year^15^. While some of it continues northward through the Socotra Channel into the Gulf of Aden^16^, most of the Somali Current deviates eastward away from the coast in two branches to form the northern limbs of anticyclonic eddies (Fig. 1a); the Southern Gyre at ~4^o^N and the Great Whirl at ~ 10^o^N^15^. These eddies advect the cooler (by up to 5 °C), nutrient-rich, upwelled water away from the coast, expanding the range of intense phytoplankton blooms over a wide area^17,18^, with the greatest chlorophyll concentrations occurring in July^12,13,19,20^.

The Somali upwelling system exhibits a complex spatial structure with multiple dynamic features at play^21^. The Southern Gyre is topographically controlled and remains at 4^o^N^22,12^, while the position of the Great Whirl is variable and controlled by the zero-wind stress curl^23^. The Great Whirl, in particular, stands out with high biological production as it extends up to 250 km across, reaching rotational velocities of up to 1 m/s^19^. In contrast, downwelling, leading to reduced productivity, occurs along the coast in between the two gyres due to the arrival of Rossby waves excited by offshore negative wind stress curl^24^. During the northeast monsoon season (December-February), the Somali Current reverses to flow southwards, meeting the northward-flowing East African Coastal Current to form the much smaller North Kenya Banks upwelling cell^25^, while the Somali upwelling subsides.

The Somali upwelling manifests significant interannual variability^26,12,13^ and seasonal variability with a stronger southwest monsoon associated with enhanced upwelling and eddy activity^12^. However, it remains unclear how it will be impacted by future climate change. For example, Bakun^27^ speculated that the future enhancement of the land-sea temperature gradient will enhance the winds and intensify global upwelling systems. However, modelling and observational results do not show a coherent response among the EBUSs, with spatial heterogeneity reported even within individual upwelling systems^6,7,28–32^. The absence of a globally coherent picture of the response of upwelling systems to climate change may result from both frequently inconsistent approaches to data analysis and the complexity and heterogeneity of mechanisms involved in the formation of upwelling systems. For instance, the length of datasets used is often inconsistent, which is particularly important for detecting decadal variability e.g., the El Niño Southern Oscillation for the Humboldt upwelling system^31^. Additionally, the spatial resolution in climate models is too coarse to resolve the fine scale features in upwelling systems, making future predictions challenging^33^.

Despite the considerable uncertainty surrounding the future fate of these systems, there is a general agreement that all the EBUSs will undergo intensification (weakening) in their poleward (equatorward) Sect.^32^. Additionally, there is evidence that the inner coastal zones of global upwelling systems may act as a buffer for surrounding background warming, which could form potential climate refugia for marine fauna^31,33–36^. However, large uncertainties remain for future changes in primary productivity, species distribution and trophic interactions^32^.

Future changes in the Somali upwelling system are also uncertain with some studies projecting a decline^6,40^ and others projecting an increase (Goes et al., 2005; deCastro et al., 2016), with recent observations finding a slight decrease in upwelling intensity from 1988 to 2018^20^ and 1958–2021^21^. Inconsistencies may be due to the complicated spatial structure of the upwelling with multiple factors e.g., the wind, the Somali Current and the anticyclonic systems, influencing the upwelling separately. For example, an increase in the coastal upwelling-favourable winds^38^ is competing with the negative wind stress curl that initiates downwelling Rossby waves further offshore^24^. The Findlater jet and Great Whirl are also projected to move northwards by the end of the century^39,40^, despite recent observations of a southward shift from 1982 to 2013^26^. Any shift in these features would lead to a subsequent spatial shift of the upwelling, which could have implications for marine ecosystem dynamics in this highly advective region.

The temperature of the tropical Western Indian Ocean is projected to rise by up to 5 °C by the end of the century under Representative Concentration Pathway (RCP) 8.5^41^. Despite this, the amount of warming is projected to be lower near the coast compared with further offshore and the rest of East Africa’s coastline^41^, indicating that the Somali coastline could also serve as climate refugia in future.

The Somali upwelling provides huge potential for domestic and foreign industrial fishers due to the high food availability for small and large pelagic fish species^42^. Small pelagic species found here include anchovies, sardines and small mackerel, while various tuna species (including skipjack, yellowfin and bigeye) and billfish dominate the large pelagic species in this region^42,43^. Future changes in the productivity of the Somali upwelling and potential abundance of these species could have consequences for both coastal communities and foreign industrial fishing fleets.

In this study, we analyse a high-resolution ocean projection extending to 2099, with a resolution of 1/4^o^—higher than most projections within the CMIP6 ensemble of Earth System Models^44^. This approach allows for a detailed examination of the Somali upwelling system with enhanced spatial granularity using the model which has undergone extensive validation for Western Indian Ocean upwelling systems^25,41,45^. However, it is essential to note that this analysis is based on a single model run which we put into the context of the CMIP6 projections. Therefore, this study aims to provide insights into understanding critical factors shaping the climate change impact on this system, rather than presenting an assessment of its future state. The latter would require an analysis of high-resolution ensemble runs, which is still beyond the computational capabilities of the ocean climate modelling community. We also utilise a size spectrum model to investigate projected changes in higher trophic levels to try and understand potential future implications on Somali fisheries and the industrial fishing fleet.

## Methods

### High-resolution ocean projection

Version 3.5 of the Nucleus for European Modelling of the Ocean (NEMO)^46^ is the physical framework used in this study and is composed of an ocean general circulation model coupled to a sea-ice model, LIM2^47^. The horizontal spatial resolution of the model is 1/4^o^, which corresponds to grid cells of 6 km at the poles and 27.8 km at the equator, with 75 vertical levels that range from 1 m to 200 m thickness at the surface and in the deep ocean respectively. To parameterize vertical mixing, a turbulent kinetic energy scheme is used^48^, which includes modifications from Madec^49^. MEDUSA-2, a plankton ecosystem model^50,51^, represents the biogeochemistry and is a size-based, intermediate complexity model where the plankton community is divided into diatoms and non-diatoms and resolves the nitrogen, iron, carbon, silicon and oxygen elemental cycles.

This NEMO-MEDUSA model configuration is forced at its surface boundary using physical output from a simulation of the HadGEM2-ES Earth system model^52,53^, performed as part of the coupled model intercomparison project phase 5 (CMIP5) for the fifth Assessment Report (AR5) of the Intergovernmental Panel on Climate Change (IPCC). Its initial conditions are derived from a ‘twin’ 1^o^ model that was spun-up from 1860 to 1975, which is adequate to allow surface ecological processes to come into quasi-equilibrium but may be too short to fully equilibrate the model^54^. This relatively short spin-up was used in order to reduce the compute costs associated with higher (1/4^o^) resolution. From 1975 to 2005, the 1/4^o^ model is run under historical atmospheric pCO_2_ concentrations and then under the IPCC RCP 8.5 pathway from 2006 to 2100. This pathway is a high emissions scenario in which CO_2_ emissions rise continuously over the 21st century and, by 2100, cause an additional 8.5 Wm^− 2^ of radiative forcing. Further details of model configuration, forcing, equilibration and verification can be found in Yool et al.^50,51,55^ and Popova et al.^56,57^.

The version of NEMO-MEDUSA used in this study has an eddy-permitting spatial resolution (1/4^o^) which is higher than that used in the majority of CMIP5 and CMIP6 models^44^ and is able to represent the main dynamical and biogeochemical features of the global ocean. In particular, it is able to represent western boundary currents and the wider ocean circulation and the upper biogeochemistry with good skill, making it a suitable tool for regional analysis^58,41^.

### Community size spectrum model

To describe the time-varying size structure of the marine ecosystem over the Western Indian Ocean, a recent implementation of the Community Size Spectrum Model (CSSM)^59^ is employed and is available at https://doi.org/10.5281/zenodo.4593394. This is a box model at its core and has been applied to large regions such as the North Sea^59^, the Agulhas Bank^60^ or Exclusive Economic Zones (EEZs)^61,62^. Here, it is used to describe the pelagic ecosystem over the wider Somali upwelling region (0-15^o^N, 40-60^o^E).

Details on the equations describing the processes in the CSSM can be found in Blanchard et al.^58,63^ and the specific model configuration used here matches prior work^60,62^ and is summarized here. The model represents depth-integrated wet mass (g/m^2^) in individuals from 1 mg to 1000 kg at 100 log-spaced size classes. Here, the focus is on the pelagic size spectrum only so demersal fish and benthic fauna are not included. Size-based preferences governs trophic interactions, which are characterized by a log-normal distribution of prey size that is centered at an optimal (median) predator: prey mass ratio of 100^59,64,65^. A size dependent-clearance rate controls food ingestion, i.e. large predators search larger volumes of water per unit time. All rates are temperature-dependent with an activation energy of 0.63 eV^61,62^. The gross growth conversion efficiency of ingested prey to predator biomass is set to 0.2^59^. This is a combination of the assimilation efficiency (proportion of ingested food that is successfully assimilated) and the loss of assimilated mass/energy to metabolic processes. Fishing is prescribed as a fixed mortality of 0.4 year-^1^ for individuals larger than 1.25 g, which accounts for fish meal production and human consumption^60,61^. This rate cannot be verified for this region due to the unreliability of catch data. The reconstructed time series of fish catch for the Somali EEZ (obtained from the Sea Around Us - see Appendix) has a reliability of score of 1-1.2 out 4, 1 being the least reliable value. This is due to few reliable national landing statistics, poorly documented illegal fishing and a substantial amount of small-scale subsistence fishing^42^, which all leads to a heavy reliance on extrapolation and assumptions to reconstruct catches.

Production of new individuals in the smallest size class (1 mg) is controlled by continuously setting the biomass in this class to an expected biomass obtained by extrapolating the plankton size spectrum^66^. Thus, the model assumes that the smallest sizes of predator will appear whenever and wherever prey is available.

Prey for the smallest size classes consists of organisms smaller than the minimum resolved size of 1 mg. This includes all unicellular plankton (bacteria, phytoplankton and microzooplankton) and most multicellular zooplankton. These fields are taken from NEMO-MEDUSA, described above. The plankton spectrum is inferred from the time-varying concentration of MEDUSA’s four plankton functional types: diatoms, non-diatom phytoplankton, microzooplankton and mesozooplankton. Each is assumed to span a fixed wet mass range of 4.2 pg–4.2 ng for non-diatom phytoplankton, 4.2 ng–4.2 µg for diatoms and microzooplankton and 10 µg–1 mg for mesozooplankton^60^. The biomass distribution of each plankton functional type within its size range is assumed to correspond to a Sheldon-Sutcliffe size spectrum, i.e. uniformly distributed over log-spaced wet mass^67^. To obtain the wet mass from nitrogen, each concentration is multiplied by MEDUSA’s C: N ratio (6.625 for phytoplankton and 5.625 for zooplankton)^50^ and an assumed wet: carbon mass ratio of 10^68^. The combined plankton spectrum contains 84 log-spaced size classes, spanning 4.2 pg-1 mg in wet mass.

In order to link with MEDUSA’s depth-resolved plankton prey, the vertical distribution of size classes in the CSSM must be known. In reality, larger organisms control their vertical position in the water column and may adjust it dynamically to predator presence and prey availability. Given that CSSM encapsulates the entire pelagic community this is challenging to represent as additional assumptions and explicit treatment of functional guilds would typically be required^69^. For example, MEDUSA’s zooplankton are simulated as simple passive tracers without any representation of vertical migration (either diel or seasonal). As such, their vertical distribution is typically from 0 to 100 m, tracking their food sources. Zooplankton can be found below this depth where deep mixing has occurred, but the majority are located in the euphotic zone. Larger organisms such as fish dynamically control their vertical position in response to prey and predators. Thus, it is assumed that the pelagic predators redistribute instantaneously in the vertical according to prey availability, for simplicity^62^. The assumption that modelled predators prefer smaller prey causes the predator concentrations to be proportional to the smallest prey, i.e., the sum of all plankton biomass at each depth. The resulting vertical predator distribution is summarized by the interaction depth, which represents the effective depth range where predator-prey interactions occur within the community. This metric depends on plankton so the depth distribution of predators will dynamically expand and contract^70^. Whilst relatively simple, this treatment of depth distribution accounts for the seasonally varying depth extent of the energy at the base of the food web, which is preferable to approaches that use a prescribed constant depth for predator distribution. In practice, this approach likely works best for smaller sizes of fish that are confined to the upper 100–200 m; it could underestimate the depth ranges of larger predators, as the absence of functional guilds in the model means features that sustain predators at depth (diurnal vertical migration, mesopelagic communities) are not represented. NEMO-MEDUSA outputs are used to compute this time-varying depth distribution, which is then used to weigh depth-resolved concentrations of plankton when computing the depth-averaged concentration.

A weighted depth-average of temperature is also derived from NEMO-MEDUSA outputs, which is based on the depth distribution of predators described above. For both temperature and plankton, depth-averaged values are extracted for each model grid box (1/4^o^ x 1/4^o^). In order to fully resolve seasonal and interannual variability, the CSSM is configured to run at daily time steps. NEMO-MEDUSA outputs are available as 5-day means and are linearly interpolated to daily means during simulation with the CSSM. Here, biomass output is stored as monthly means. Total biomass across all size classes and biomass for fish < 250 g and > 250 g are analysed separately and arbitrarily represent small (e.g., sardines tuna anchovies) and large pelagic (e.g., billfish and tuna) fish species [42; 43]. However, it must be noted that small individuals of large pelagic species may fall into the small pelagic category.

### CMIP6 simulations

**Fig. 2:**
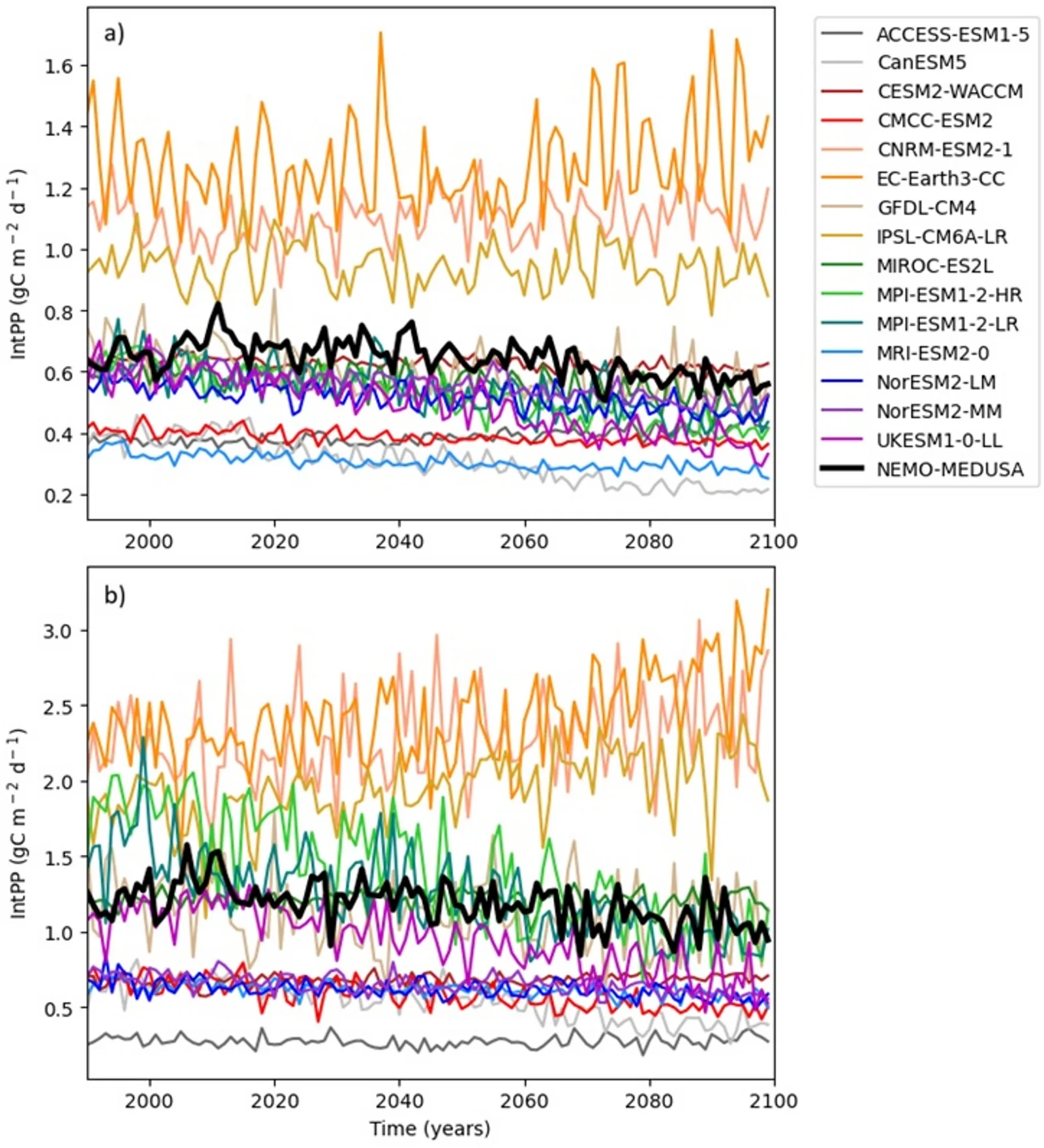
IntPP (**a**) annual mean and (**b**) July time series for the Somali upwelling region (48-55^o^E, 5.5-12.5^o^N) for 15 CMIP6 models under the SSP 5-8.5 scenario and the NEMO-MEDUSA simulation following RCP8.5. Note the different scales on the y axis. CESM2-WACCM does not appear in (**b**) as monthly output was not accessible for SSP 5-8.5.

In order to assess where the NEMO-MEDUSA projection belongs in the multi-model ensemble for the Somali upwelling and wider Indian Ocean, biological production is assessed in 15 CMIP6 simulations^71^. Monthly integrated primary production (variable IntPP in CMIP6) under the historical (2000–2009) simulations are used to calculate annual mean IntPP and July IntPP (peak Somali upwelling season) over the tropical Indian Ocean. Time series of annual and July IntPP are also calculated over the Somali upwelling region (48-55^o^E, 5.5-12.5^o^N) from 2000 to 2100 under SSP5-8.5 scenario. The CMIP6 models used can be seen in Fig. 2.

### Observational datasets for validation

To evaluate the skill of the ‘present-day’ state of the model, satellite-derived observations of the surface circulation, sea surface temperature and chlorophyll-a are utilized over the wider Somali upwelling region from 5^o^S to 15^o^N, 35^o^E to 60^o^E.

To assess the surface circulation, the absolute geostrophic velocities are used from the satellite altimetry product AVISO (distributed by the Copernicus Marine Servicehttps://data.marine.copernicus.eu/product/SEALEVEL_GLO_PHY_L4_MY_008_047/description. Specifically, geostrophic zonal and meridional velocities that are computed weekly on a ¼^o^ x ¼^o^ grid from the delayed time ‘Update’ DUACS-DT2018 version. Here, the velocities are averaged in July from 2000 to 2020.

The reprocessed SST Met Office Operational-Sea-Surface-Temperature-and-Sea-Ice-Analysis (OSTIA) global product spans from 1981 to 2021 and is available at 5 km spatial resolution (http://marine.copernicus.eu/services-portfolio/access-to-products/*).* To assess modelled chlorophyll-a, satellite observations were taken from the ESA Ocean Colour CCI (OC-CCI; v3.1; http://www.esa-oceancolour-cci.org/), which is a global dataset that stores monthly means at 4 km spatial resolution. It is one of the most complete, multi-sensor datasets of ocean colour that is currently available and is widely validated^72^. The July average from 2000 to 2020 is used to evaluate the modelled SST and chlorophyll-a in the Somali upwelling region.

To compare IntPP in NEMO-MEDUSA and the suite of CMIP6 models with observations, a simple average is calculated from three satellite-derived estimates following Popova et al.^58^ and Jacobs et al.^41^; the Vertically Generalized Production Model (VGPM)^73^, the Eppley-VGPM^74^ and the Carbon-based Production Model (CbPM)^75^.

## Results

### Model validation

The altimetric sea surface currents show the fast, northward-flowing, Somali Current along the Somali coast during the southwest monsoon season (shown as the July multidecadal mean; Fig. 1a), with velocities exceeding 1 m/s. The anticyclonic gyres are visible with the current deviations from the coast at 4^o^N for the Southern Gyre and 10^o^N for the Great Whirl also evident. There is strong qualitative agreement between the altimetric and modelled surface currents (Fig. 1b) with the position and speed of the Somali Current captured well in the model. The model is also able to simulate the Great Whirl with the deviation from the coast clearly evident at 10^o^N. However, the smaller deviation from the Southern Gyre is not captured as well in the model. Additionally, differences at the equator are due to the geostrophic assumption not being valid for the altimetric currents.

The imprint of the Somali upwelling is revealed in the multidecadal average for SST and surface chlorophyll-a in the observations (Fig. 1c and e) with cooler (< 25 °C) waters and elevated chlorophyll-a concentrations (> 1 mg/m^3^) evident along the coast and along the northern edge of the Great Whirl (also evident in the model – see Fig. 1d and f). Despite the Somali Current exhibiting greater velocities and the wider region being slightly warmer the model adequately simulates the physical characteristics of the Somali upwelling. Greater disparities occur for chlorophyll-a concentrations, which is overestimated along the northern edge of the Great Whirl in the model (Supplementary Fig. 1). However, the spatial pattern of elevated concentrations along the Somali coast and along the Great Whirl’s northern edge is captured by the model. It should also be noted that due to the coarse resolution of the atmospheric forcing fields (1^o^), the wide drop-off of the wind forcing reduces baroclinic instabilities and may limit the subduction of nutrients below the euphotic zone^76^. Consequently, this may overestimate productivity along the coast in the model.

The spatial pattern and magnitude of IntPP in the tropical Indian Ocean is highly variable across the CMIP6 models for both the annual and July mean (Supplementary Figs. 2 & 3). NEMO-MEDUSA is broadly consistent with the satellite-derived product but underestimates IntPP in the open ocean, comparable with other CMIP6 simulations. The spatial pattern of the productivity induced by the Somali upwelling is captured well during its peak in July (Supplementary Fig. 4), which is not the case for some CMIP6 models.

For the Somali upwelling region, overall there is a large spread in the projected changes in IntPP until the end of the century with no clear concensus on the trend or magnitude of interannual variability across all models (Fig. 2). This is consistent with findings for the wider Indian Ocean^71,77^. 12/16 models, including NEMO-MEDUSA project a significant, decline in annual average IntPP by the end of the century (Fig. 2a; Supplementary Table 1). The projections for July are more inconsistent. NEMO-MEDUSA projects a slight decline in IntPP in line with a number of other models (e.g., GFDL, MIROC, MPI, MRI, NorESM2 and UKESM) with the IntPP magnitude falling in the middle of CMIP6 simulations. Thus, NEMO-MEDUSA is broadly consistent with the large-scale features of this region in CMIP6 models but provides additional higher resolution details of the Somali upwelling system. This is further evidenced by a comparison with NEMO-MEDUSA’s twin 1^o^ simulation (Supplementary Fig. 5), which shows substantial improvements in the high-resolution 1/4^o^ simulation. Specifically, the position and extent of the Somali Current, with the 1^o^ run unable to simulate the Great Whirl. Consequently, this translates to the 1^o^ run also being unable to accurately simulate the cooler, productive waters generated by the presence of the Great Whirl, which is better captured by the 1/4^o^ simulation.

### Projected changes in the Somali Current

**Fig. 3:**
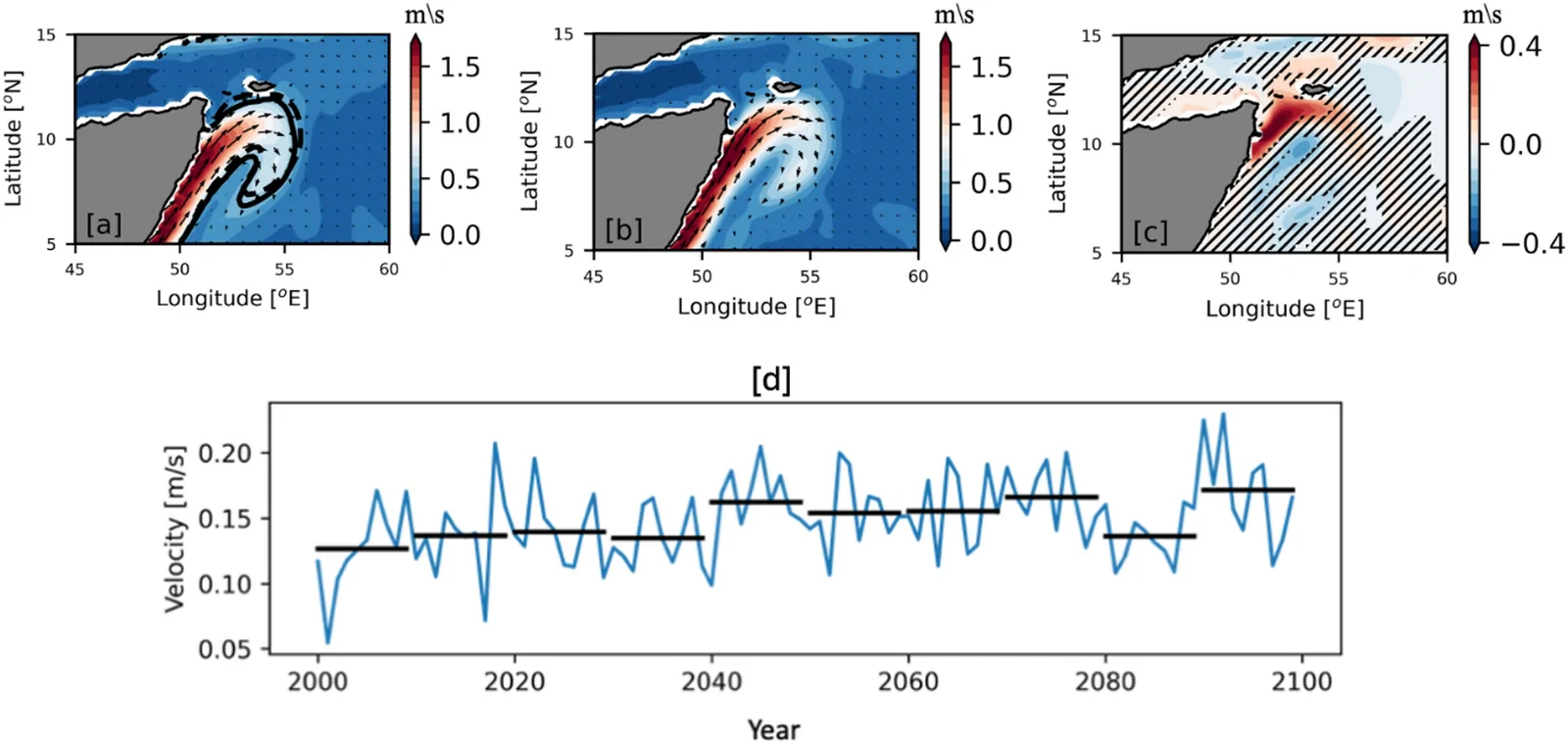
Multidecadal mean (July) surface current speed (m/s) for 1993–2020 [**a**] and 2070–2099 [**b**], anomalous surface currents speed for the late century period minus the early century period [**c**]. The dashed contours on [**a**] are the surface current speed contours of 0.5 m/s for the early century period (black line) and late century period (dashed line). [**d**] is the mean surface current speed across 12^o^N through the Socotra Channel from 51-55^o^E, black lines indicate decadal means. The hatched area on [**c**] represent anomalies that are not significant at the 95% confidence interval, after performing a statistical t-test.

Changes in ocean currents are a key climate stressor for marine ecosystems^78,41^ and as the Somali Current plays an important role in driving the upwelling, any changes could have major impacts on the region’s productivity. The projected mean surface currents in July, i.e., at the peak of the upwelling season (Supplementary Fig. 4), for the beginning (1993–2020) and end of the century (2070–2099) are shown in Fig. 3a and b respectively, with the end of century anomaly shown in Fig. 3c. By the end of the century, the Somali Current is projected to strengthen north of 9^o^N and shift northwards, changing the position of the Great Whirl. This is evident by the negative anomaly from 9-10^o^N (0.2 m/s) and the positive anomaly (> 0.4 m/s) immediately to the north (Fig. 3c). The northward shift of the core of the current, here defined by the 0.5 m/s contour, can be seen more clearly in Fig. 3a, with the projected position at the end of century (dashed line) situated further north than its position at the beginning of the century (solid line). In addition to the northward shift, the anomalies are greater on the northern side, which indicates a projected increase in the areal extent of the Somali current.

This northward shift has led to enhanced flow through the Socotra Channel into the Gulf of Aden, with current velocities across 12^o^N steadily increasing from 2000 to 2099 (Fig. 3d). By the 2090s, the velocity through this channel is projected to be 35% greater compared with that in the 2000s, which could have consequences for marine ecosystems, e.g., loss of nutrients out of the region and changes in connectivity affecting larval dispersal^79^.

**Fig. 4:**
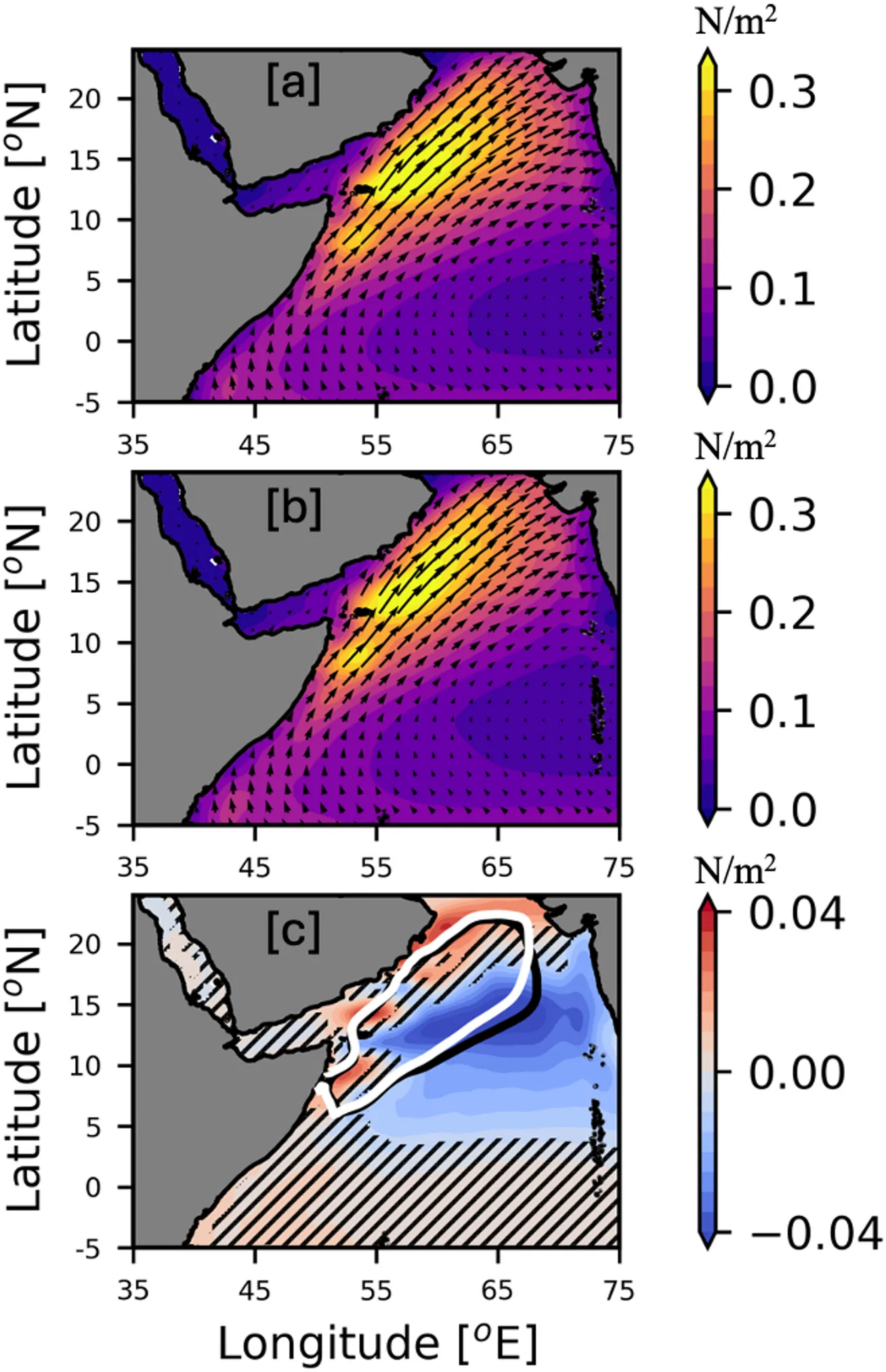
Multidecadal mean (July) wind stress (N/m^2^) for 1993–2020 [**a**], 2070–2099 [**b**] and anomalous wind speed for the late century period minus the early century period [**c**]. 9 m/s contours on [**c**] for 1993–2020 (black) 2070–2099 (white). The hatched area on [**c**] represent anomalies that are not significant at the 95% confidence interval, after performing a statistical t-test.

During the southwest monsoon, the Somali Current is driven by the southwesterly winds across the northwest Indian Ocean (Fig. 4a), which are responsible for its strengthening during this time of year^15^. The projected northward shift of the Somali Current could be caused by the northward shift in these winds by the end of the century (Fig. 4b). This is further evidenced in Fig. 4c, which shows the anomalous wind speed during 2070–2099 (compared with 1993–2020), with projected reductions in wind speed of up to 2 m/s across the basin from the equator to ~ 20^o^N and increases projected to the north of this. The white line on Fig. 4c illustrates the northward shift in the 9 m/s winds, which are most apparent, and statistically significant, north of 5^o^N.

### Projected changes in productivity & fisheries

**Fig. 5:**
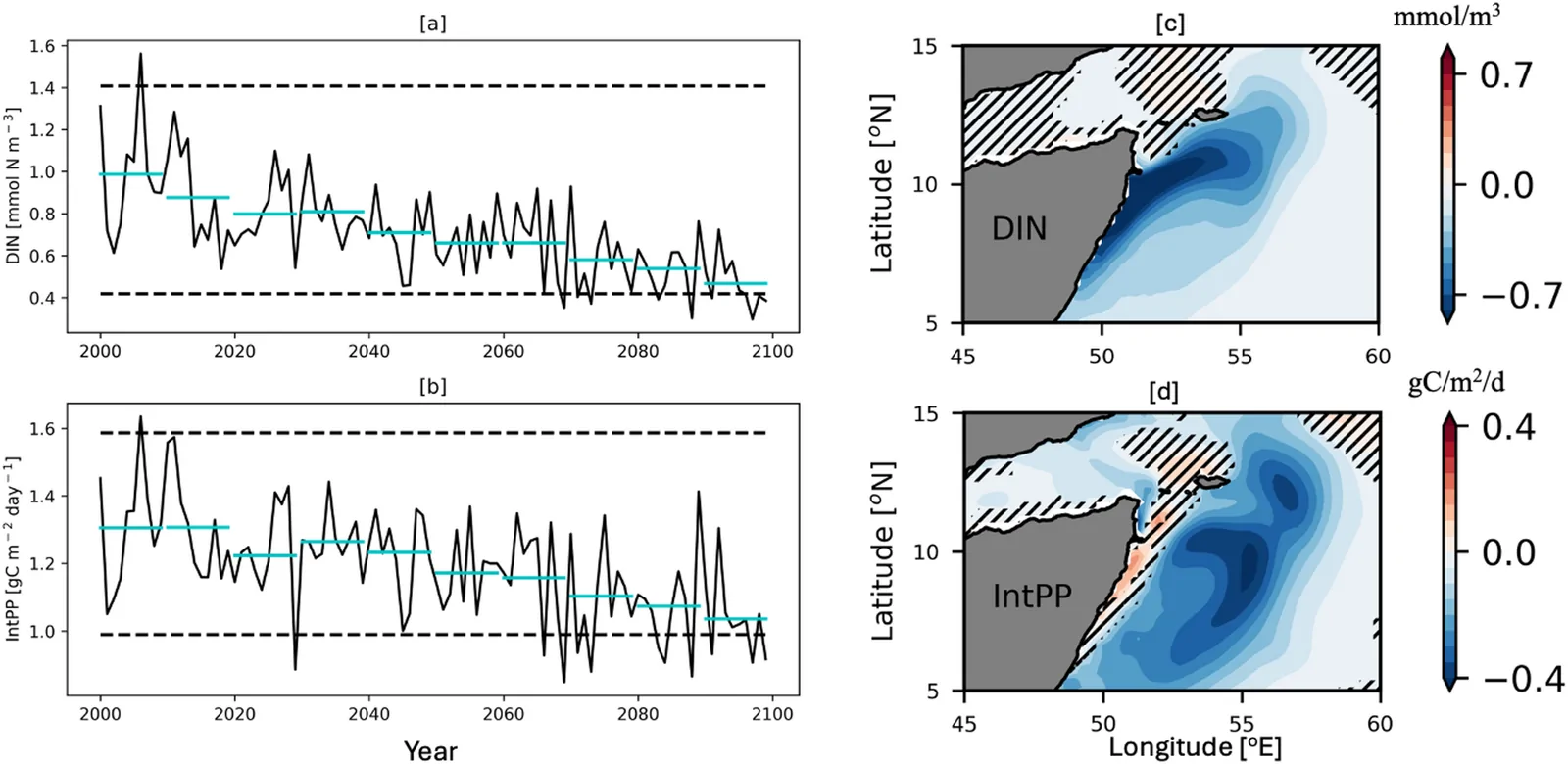
[**a**] Mean dissolved inorganic nitrogen (DIN; mmol/m^3^) and [**b**] integrated primary production (IntPP; gC/m^2^/d) in July over the Great Whirl region (48–55^o^E, 5–12^o^N), and anomalous DIN [**c**] and IntPP [**d**] for 2070–2099 minus 1993–2020. Turquoise lines on [**a**, **b**] are decadal means and black lines represent 1 standard deviation from 1993–2020. The hatched area on [**c**, **d**] represent anomalies that are not significant at the 95% confidence interval, after performing a statistical t-test.

Changes in nutrient supply and, consequently, productivity are also major drivers for marine ecosystems and could have severe implications for regional fisheries, particularly in areas dominated by upwelling. At the start of the century (2000s), dissolved inorganic nitrogen (DIN) concentrations and integrated primary production (IntPP) are elevated in the Somali upwelling region, particularly close to the coast and in the vicinity of the Great Whirl with DIN and IntPP reaching 2 mmol/m^3^ and 2 gC/m^2^/d respectively (Supplementary Fig. 6). Averaged over the Great Whirl (from 48-55^o^E, 5-12^o^N) in July, DIN concentrations are projected to decline by 47% by the 2090s (Fig. 5a), while a 26% decline is projected for IntPP (Fig. 5b). For both parameters, values are projected to regularly fall outside their natural range of variability (i.e., exceed 1 standard deviation from baseline period 1993-20) from the 2060s. It should be noted that similar declines are projected for the entire southwest monsoon season, but the decline is largest during July (Supplementary Fig. 4).

These projected reductions are evident over the wider Great Whirl region by the end of the century (Fig. 5c and d). The largest decline in DIN concentrations (> 0.8 mmol/m^3^) is projected to occur in the path of the Somali Current and northern branch of the Great Whirl, indicating reduced upwelling and advection of nutrients further east. This is projected to cause a widespread reduction in IntPP (up to 0.4 gC/m^2^/d) further offshore from the eastern coast of Somalia. In contrast, there is a projected increase of up to 0.1 gC/m^2^/d along the coast and beyond Socotra Island from ~ 8-13^o^N. This is concurrent with the anomalous increase in surface speed of the Somali Current, which could indicate increased upwelling due to dynamic uplift^45^. However, it could also partly be a consequence of the model overestimating coastal productivity due to the coarse resolution of wind forcing (outlined in 3.1).

**Fig. 6:**
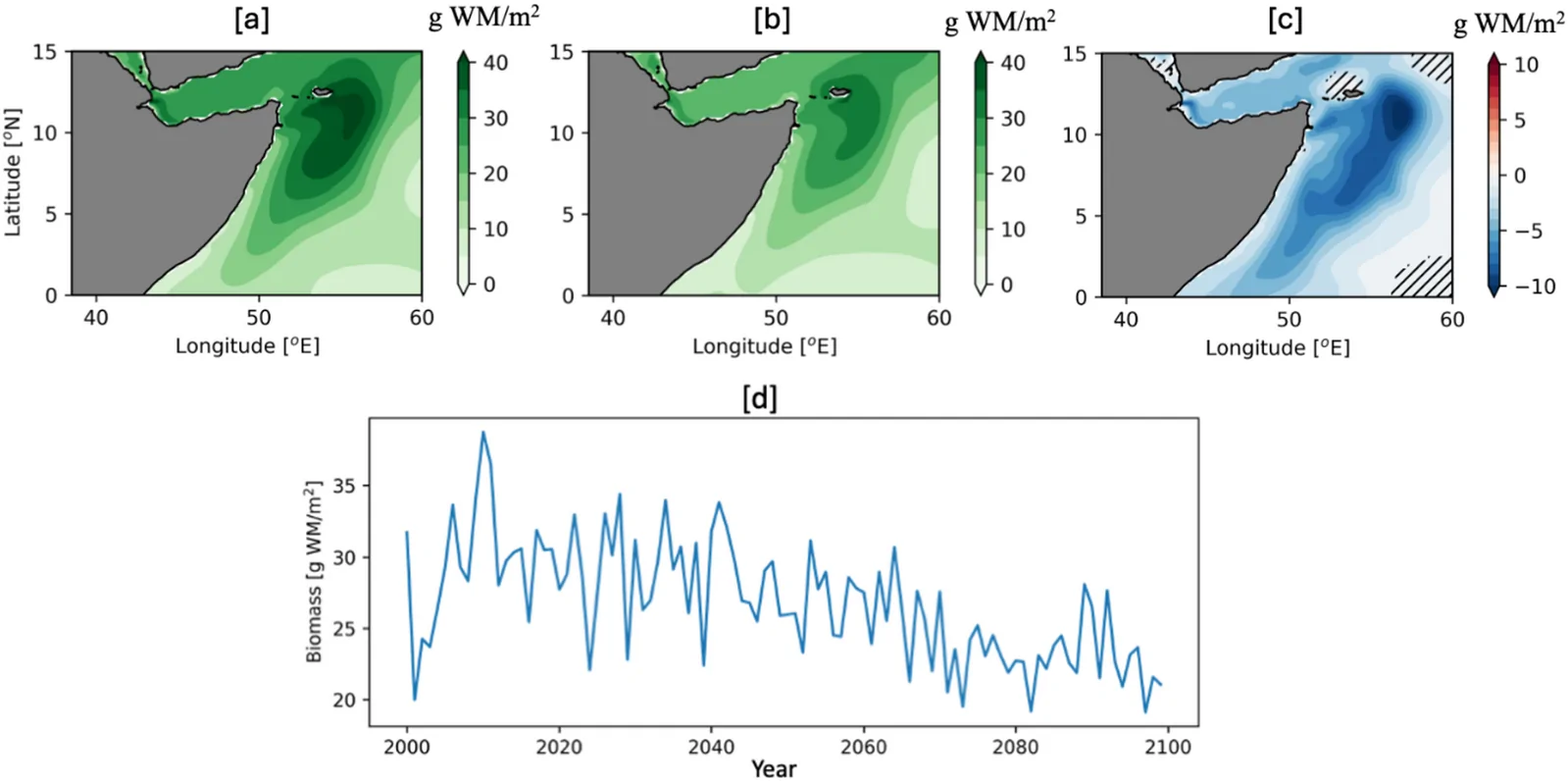
Multidecadal mean (July) total biomass (g WM/m^2^) for 1993–2020 [**a**], 2070–2099 [**b**] and anomalous total biomass for the late century period minus the late century period [**c**]. The hatched area on [**c**] represent anomalies that are not significant at the 95% confidence interval, after performing a statistical t-test. Mean total biomass in July over the Great Whirl region (48-55^o^E, 5-12^o^N) [**d**].

To explore the potential impact a reduction in IntPP may have on the region’s fisheries a community size spectrum model was employed to assess any changes in higher trophic levels. Figure 6a shows the total biomass across all size classes averaged from 1993 to 2020, which exceeds 40 g WM/m^2^ (WM: wet mass) in the vicinity of the Great Whirl and Socotra Island. By the end of the century (Fig. 6b), the total biomass is projected to decline by up to 10 g WM/m^2^ across the Somali Upwelling region, Great Whirl and east of Socotra Island (Fig. 6c). However, there is a narrow band along the eastern Somali coast from ~ 3-10^o^N, approximately where IntPP is projected to increase, that is projected to undergo little to no decline in total biomass, which could indicate a potential refuge area. Despite this, total biomass is projected to decline by ~ 20% in the Great Whirl region by the end of the century, with a steady decline indicated from the 2040s (Fig. 6d).

**Fig. 7:**
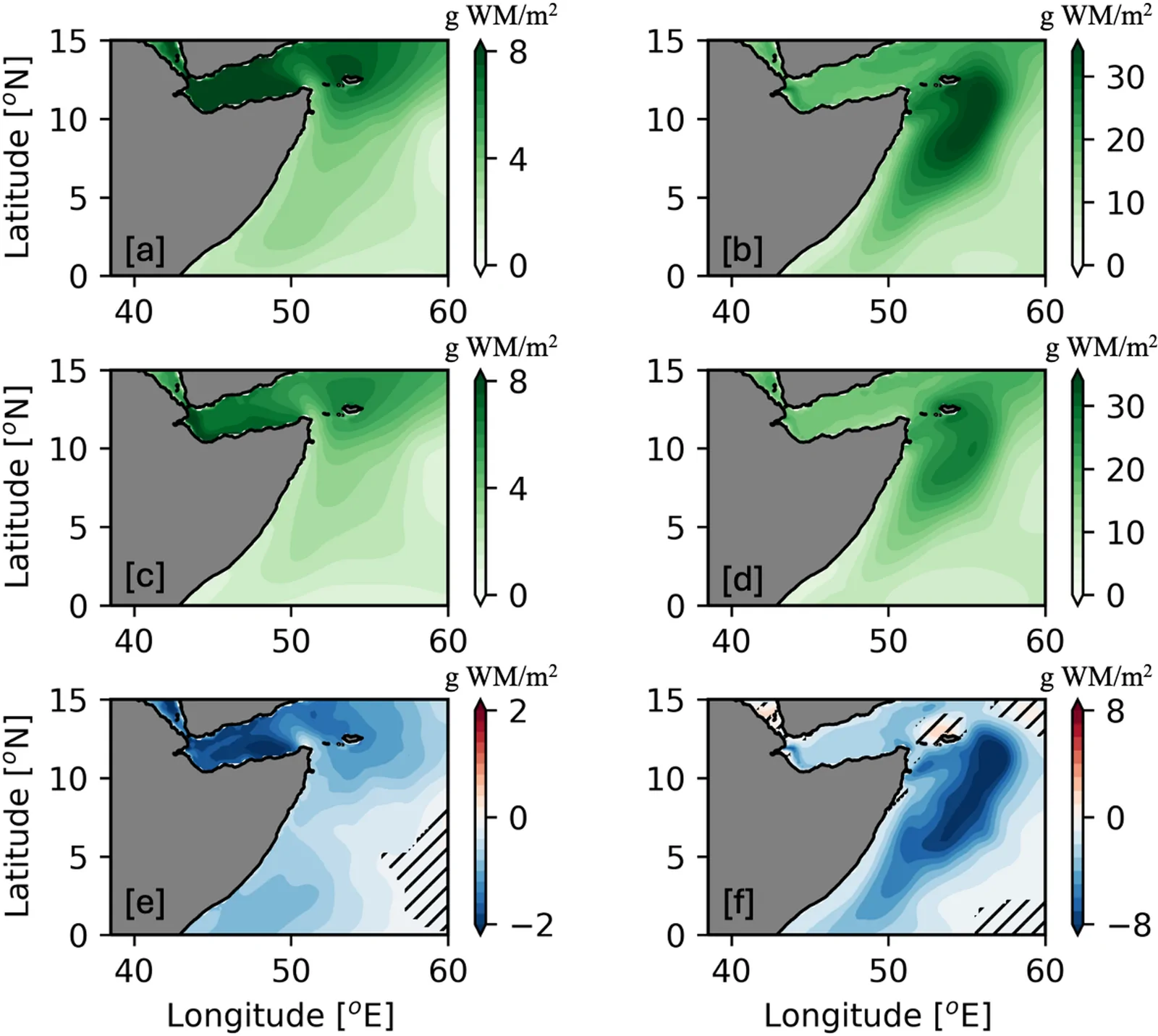
Multidecadal mean (July) biomass (g WM/m^2^) for large fish (> 250 g) [**a**, **c**] and small fish (< 250 g) [**b**, **d**] for 1993–2020 [**a**, **b**] and 2070–2099 [**b**, **d**], and anomalous biomass for the late century period minus the early century period for large fish [**e**]. and small fish [**f**]. The hatched area on [e, f] represent anomalies that are not significant at the 95% confidence interval, after performing a statistical t-test.

Distinguishing the total biomass between small fish (< 250 g) and large fish (> 250 g) reveals that, while small pelagics dominate everywhere, the Gulf of Aden and Socotra Island see a greater proportion of large fish (Fig. 7a) than the Great Whirl region, which is clearly dominated by smaller fish (Fig. 7b) during the early century. By the end of the century (Fig. 7c and d), both large and small fish are projected to decline, consistent with the decline in total biomass (Fig. 6). In the Great Whirl and wider Somali upwelling region, this decline is projected to be greatest for smaller fish with a > 8 g WM/m^2^ reduction projected across the region, which indicates a maximum of > 40% decline (Fig. 7f). In contrast, large fish are projected to have the greatest decline in the Gulf of Aden and around Socotra Island (~ 25%; Fig. 7e).

The spatial distribution of small fish and their decline under climate change generally follow the modelled distribution of zooplankton (Supplementary Fig. 7). The latter shows a peak downstream of the nearshore maximum in DIN and productivity, reflecting the combined effects of advection and the time lag between increases in primary producers and subsequent zooplankton growth. The area of greatest projected loss of small fish biomass is also coincident with the greatest projected warming (Supplementary Fig. 8). In contrast, the spatial distribution of large fish does not correspond to the season-specific patterns of phyto- and zooplankton as it is shaped more by integrated ecosystem conditions and life-history traits than by immediate, seasonal variations in lower trophic levels.

**Fig. 8:**
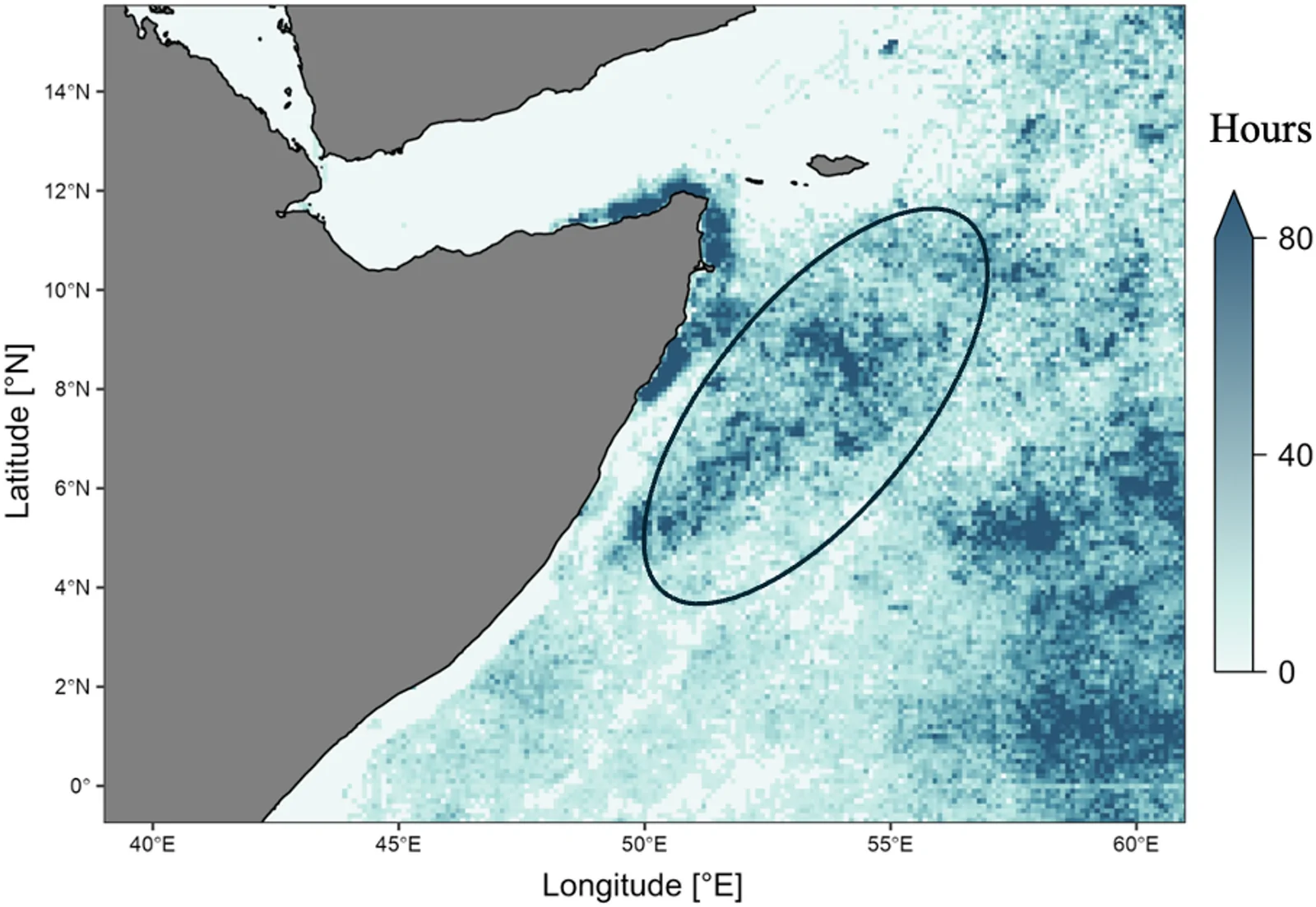
Total Apparent fishing effort (hours) in 2023 in 0.1 × 0.1° resolution grid cells. Sourced from Global Fishing Watch (https://globalfishingwatch.org/).

In summary, under RCP8.5, there is a projected decline, but not a total collapse, in biomass over the Great Whirl region by ~ 20% by the end of the century. Here, and over the wider Somali upwelling region, this decline is dominated by smaller fish (< 250 g), with projected reductions of > 40% in some places. This could substantially impact the international fishing fleets that target the productivity generated by the Somali upwelling, as the largest vessel presence (aside from the coastal region) is located over the region experiencing the largest declines, with data from 2023 shown as an example in Fig. 8. It is unclear whether this will affect the small-scale fishing fleet, which are less likely to be equipped with Automatic Identification Systems (AIS) trackers and are not included in this dataset.

## Discussion

Understanding how the world’s major upwelling systems will be impacted by anthropogenic climate change remains a challenge. There is still no consensus on how these systems will be affected, with varying results between systems, even within the same system, and between different observational datasets and models. Given their potential as climate refugia for commercially important species, it is vital to understand any potential changes to ensure adequate mitigation measures are put in place to protect coastal communities’ livelihoods and emerging blue economies.

Unlike the EBUSs, that are active year-round, the Somali upwelling system is unique and is the largest of the global ocean’s seasonal upwelling systems^80^, presenting an even greater challenge. Here, we assessed the future projection from a high-resolution (1/4^o^) global ocean model, which is more appropriate to evaluate dynamical systems at the regional and seasonal scale than the coarser resolution (up to 1^o^) CMIP5 and CMIP6 models^44^. This improvement in accuracy of the currents, SST and chl-a is also evidenced by the comparison with NEMO-MEDUSA’s 1^o^ twin simulation (Supplementary Fig. 5) and likely improves the reliability of the future projection.

Our analysis focuses on the month of July, i.e., the peak of the southwest monsoon season (Supplementary Fig. 4), and under a high emissions scenario (RCP8.5), the Somali upwelling is projected to decline. The key projected changes by the end of the century are discussed below.

The Somali Current and Great Whirl are projected to shift northward by up to 0.5^o^ by the end of the century, which is likely induced by a northward shift in the prevailing southwesterly winds. The projected shift in the wind field is consistent with other studies using both CMIP5 and regional models^39,40^. Subsequently, this causes increased flow through the Socotra Channel, with surface velocities of > 35% greater by the 2090s. This could lead to a loss of nutrients and an increase in larval dispersal^79^, which has implications for marine ecosystems along the Somali coast and further north in the Gulf of Aden and Arabian Sea.

The changes in the wind and Somali Current may also lead to increased coastal upwelling north of 8^o^N, indicated by slightly greater productivity projected along the coast of Somali and by Socotra Island. This is in agreement with De Castro et al.^9^ and Praveen et al.^40^ who found enhanced upwelling along the same stretch of coastline by the end of the century. Both studies found that this was mainly due to increased Ekman transport induced by stronger winds, which concurs with Bakun’s^80^ theory that larger temperature differences between the land and ocean will lead to increased coastal upwelling. In addition to enhanced wind, here we show a northward shift in the Somali Current, which may be inducing additional upwelling along the coast due to dynamic uplift^45^, and in the Great Whirl, which causes a northward shift in the deviation of the current away from the coast.

The phytoplankton blooms in the vicinity of the Great Whirl are projected to decline (47% decline in DIN concentrations and 26% decline in IntPP) by the end of the century. The improvement in the 1/4^o^ model’s ability to simulate the Somali current and Great Whirl, compared with the 1^o^ CMIP6 models, likely enables greater accuracy in the projected changes in IntPP, due to high productivity occurring in its vicinity. This results in a total biomass decline in the higher trophic levels by ~ 20%, which is dominated by small (< 250 g) fish and the largest losses (up to 40%) are coincident with an area dominated by foreign industrial fishing fleets. This contrasts with the Gulf of Aden, which, compared with the Great Whirl region, shows a greater projected decline of large pelagic (> 250 g) fish. The projected decline in biomass concurs with Cheung et al.^62^ who found a projected decline in maximum catch potential by up to 40% is likely to occur in the tropics over the course of the century.

The industrial fishing fleets that operate over the wider Somali upwelling area tend to focus on tuna, which are larger, migratory pelagic fish. However, tuna distribution is associated with primary productivity abundance^82^, and thus, they are commonly found near small pelagics. The combination of projected declines in productivity, small pelagics and the exceedance of their thermal limits^41^ suggests that this region may become unsuitable habitat for tuna towards the end of the century. Even if they are able to adapt by residing in deeper water or changing their migration patterns, this will likely require the involvement of governing bodies, e.g. the Indian Ocean Tuna Commission, to effectively regulate this fishery^82^.

This contrasts with the projected increase in productivity near the Somali coast and the minimal change in total biomass, which suggests that the artisanal fishing fleet, dominated by coastal communities reliant on pelagic fish^42^, will be largely unaffected by changes in productivity. Additionally, projections indicate that warming near the Somali coast will be lower than the rest of the East African coastline and further offshore^41^. This, combined with the minimal changes in productivity, indicate that this region could act as climate refugia, in line with other global upwelling systems^31,36^. However, the lack of information on present-day Somali fisheries makes it extremely challenging to try and predict how they might be affected by future climate change. Projects like the Fisheries and Marine Ecosystem Modelling Intercomparison Project (FishMIP)^83^, are vital to help understand the impact of climate change on fisheries and marine ecosystems to be able to better inform policy, particularly for data sparse regions like that impacted by the Somali upwelling.

Despite increasing evidence that upwelling systems are likely to serve as climate refugia in future, large uncertainties remain for the upwelling strength, productivity and species distribution as a whole^32^. It is difficult to get a unified view of how the world’s upwelling systems will be impacted by future climate change because each system has a complex spatial structure that is driven by local or regional processes that lead to heterogeneous responses. The Somali upwelling system, in particular, is driven by competing factors, making it a challenge to disentangle the changes to each mechanism and understand how the system will be impacted as a whole. Other upwelling systems, such as the EBUSs, cover a vast area, with future climate change impacts projected to vary within each system^32^. Thus, when considering climate change adaptation, it is important to consider each system as a combination of multiple zones, each driven by different local or regional mechanisms. This, combined with the fact that CMIP6 models remain inadequate and generally overestimate SST for these systems^84^, highlights the need to employ models of the highest spatial resolution available to be able to resolve the mesoscale dynamics of individual systems.

## Conclusions

We use a high-resolution (1/4°) coupled physics-biogeochemistry ocean model to understand how climate stressors will impact the seasonal Somali upwelling system and its marine ecosystems over the coming century. Under a high-emissions scenario (RCP8.5), the coastal Somali Current and the offshore Great Whirl are projected to shift northwards during the height of the southwest monsoon, coincident with a northward shift in the prevailing southwesterly winds. Overall, productivity generated by the Somali upwelling is projected to decline (-26%) with evidence that the inner coastal zone, north of 8°N, will experience elevated productivity by the end of the century. These patterns suggest that, by operating with the relative climate refugia of the inner coastal zone, the artisanal fishing fleet may experience limited change, while the industrial fishing fleet, currently operating in the offshore Great Whirl region, may be more significantly impacted. Our study underscores that global upwelling systems will likely be impacted by future climate change, but that predicting the detail of this change remains challenging, with the result that models of the highest resolution must be employed to best understand these systems and enable effective mitigation to ensure the resilience of coastal communities.

## Supplementary Information

Below is the link to the electronic supplementary material.

Supplementary Material 1

## Figures and Tables

**Fig. 9:**
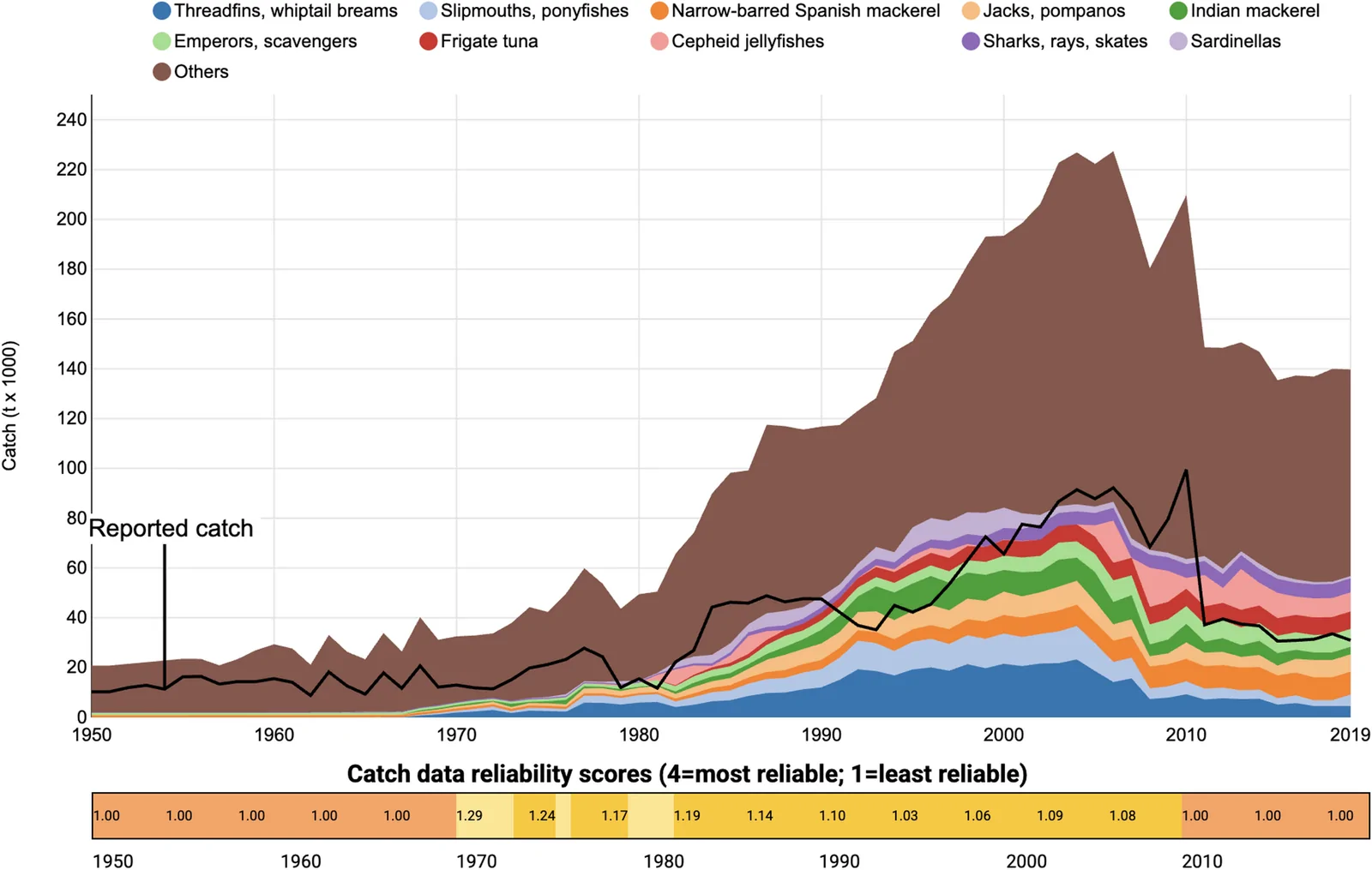
Reconstructed catch data segregated by species averaged over the Somali EEZ with the respective reliability score indicated beneath the time series. This data is retrieved from the Sea Around Us at https://www.seaaroundus.org/data/#/eez/706?chart=catch-chart&dimension=taxon&measure=tonnage&limit=10.

## Data Availability

The model output from NEMO-MEDUSA and all CMIP6 projections are available on the CEDA portal at [https://archive.ceda.ac.uk/](https:/archive.ceda.ac.uk) . The CSSM model output is available from the corresponding author on request. The satellite products used in the study can be accessed at [](http:/marine.copernicus.eu/services-portfolio/access-toproducts/?option=com_csw&view=details&product_id=SEALEVEL_GLO_PHY_L4_REP_OBSERVATIONS_008_047)[https://data.marine.copernicus.eu/product/SEALEVEL\_GLO\_PHY\_L4\_MY\_008\_047/description](https:/data.marine.copernicus.eu/product/SEALEVEL_GLO_PHY_L4_MY_008_047/description) and [http://www.esa-oceancolour-cci.org/](http:/www.esa-oceancolour-cci.org) . The data on apparent fishing effort can be accessed at https://globalfishingwatch.org.

## Appendix

See Figure 9.
