# Supplementary material for: The future fate of Somali upwelling productivity and the implications for fisheries under climate change
Source: Sci Rep. 2026 Jun 17;16:27734. doi: 10.1038/s41598-026-55455-3 (PMC13542134; doi:10.1038/s41598-026-55455-3)
Supplement: Supplementary file 1 [file 41598_2026_55455_MOESM1_ESM.docx]

**Supplementary Figures**

Supplementary Figure 1: Difference between modelled and observed surface currents [a], SST [b] and chl-a [c] in July over the wider Somali upwelling region, averaged from 2000-2020.

Supplementary Figure 2: Annual mean IntPP for 2000-2009 in the Indian Ocean for 15 CMIP6 models, the NEMO-MEDUSA simulation and the mean of three satellite products (VGPM, Eppley and CBPM).

Supplementary Figure 3: July mean IntPP for 2000-2009 in the Indian Ocean for 15 CMIP6 models, the NEMO-MEDUSA simulation and the mean of three satellite products (VGPM, Eppley and CBPM).

Supplementary Table 1: Results of Mann-Kendall statistic test to determine whether there is a monotonic trend for the annual and July timeseries in Figure 2. The trend, slope and p value are reported for each CMIP6 model and the NEMO-MEDUSA model.

| Model | Annual trend | | | July trend | | |
| --- | --- | --- | --- | --- | --- | --- |
|  | Trend | Slope | p | Trend | Slope | p |
| NEMO-MEDUSA | Decreasing | -0.0011 | <0.001 | Decreasing | -0.0022 | <0.001 |
| ACCESS-ESM1-5 | Increasing | 0.0005 | <0.001 | No trend | <-0.0001 | 0.761 |
| CanESM5 | Decreasing | -0.0020 | <0.001 | Decreasing | -0.0034 | <0.001 |
| CESM2-WACCM | Decreasing | -0.0003 | <0.001 | Increasing | 0.0004 | <0.001 |
| CMCC-ESM2 | Decreasing | -0.0004 | <0.001 | Decreasing | -0.0021 | <0.001 |
| CNRM-ESM2-1 | No trend | 0.0005 | 0.06 | Increasing | 0.0026 | 0.002 |
| EC-Earth3-CC | No trend | -0.0014 | 0.158 | Increasing | 0.0049 | <0.001 |
| GFDL-CM4 | Decreasing | -0.0014 | <0.001 | Decreasing | -0.0021 | 0.004 |
| IPSL-CM6A-LR | No trend | -0.0003 | 0.221 | Increasing | 0.0042 | <0.001 |
| MIROC-ES2L | Decreasing | -0.0008 | <0.001 | No trend | 0.0001 | 0.495 |
| MPI-ESM1-2-HR | Decreasing | -0.0025 | <0.001 | Decreasing | -0.0096 | <0.001 |
| MPI-ESM1-2-LR | Decreasing | -0.0019 | <0.001 | Decreasing | -0.0061 | <0.001 |
| MRI-ESM2-0 | Decreasing | -0.0006 | <0.001 | Decreasing | -0.0008 | <0.001 |
| NorESM2-LM | Decreasing | -0.0009 | <0.001 | Decreasing | -0.0009 | <0.001 |
| NorESM2-MM | Decreasing | -0.0012 | <0.001 | Decreasing | -0.0012 | <0.001 |
| UKESM1-0-LL | Decreasing | -0.0027 | <0.001 | Decreasing | -0.0056 | <0.001 |

Supplementary Figure 4: Seasonal cycle of [a] DIN (mmol m^−3^) and [b] IntPP for the 2000s and the 2090s over the Great Whirl region (48-55^o^E, 5-12^o^N) in NEMO-MEDUSA.

Supplementary Figure 5: NEMO-MEDUSA multidecadal mean (2000-2020) [a, b] surface currents (m/s), [c, d] SST (^o^C) and [e, f] chl-a (mg/m^3^) in July over the wider Somali upwelling region for the 1^o^ (left) and 1/4^o^ (right) simulations.

Supplementary Figure 6: Mean (July) DIN in 2000s [a] and 2090s [b], and mean (July) IntPP in 2000s [c] and 2090s [d] over the wider Somali upwelling region.

Supplementary Figure 7: Multidecadal mean (July) of depth-integrated mesozooplankton (mmol-N/m^2^) for 1993-2020 [a] and 2060-99 [b], and microzooplankton (mmol-N/m^2^) for 1993-2020 [d] and 2060-99 [e], with their respective anomalies for the late century period minus the early century period for mesozooplankton [c] and microzooplankton [f]. The hatched area on [c, f] represent anomalies that are not significant at the 95% confidence interval, after performing a statistical t-test.

Supplementary Figure 8: Multidecadal mean (July) of sea surface temperature (SST; ^o^C) for 1993-2020 [a] and 2060-99 [b], and the SST anomaly for the late century period minus the early century period. The hatched area on [c] represents anomalies that are not significant at the 95% confidence interval, after performing a statistical t-test.
